# Multidisciplinary Team Approach for Managing Pancreatic Cysts: Impact, Bias, and Opportunities for Improvement

**DOI:** 10.1002/ueg2.70243

**Published:** 2026-06-04

**Authors:** Tom Konikoff, Shounak Majumder

**Affiliations:** ^1^ Division of Gastroenterology and Hepatology Mayo Clinic Rochester Minnesota USA

Pancreatic cystic lesions (PCLs) are increasingly detected on imaging, with prevalence rising with age [1, 2]. Although most are benign, a subset, particularly intraductal papillary mucinous neoplasms and mucinous cystic neoplasms, carry malignant potential and therefore require careful evaluation, risk stratification and, sometimes, surgical resection. Although diagnostic tools and management guidelines exist, identifying high‐grade dysplasia and invasive cancer (advanced neoplasia) in PCLs and selecting patients for resection remain imperfect. As a result, management decision‐making remains challenging, particularly as incidental PCL detection continues to rise. Multidisciplinary team (MDT) discussions serve as a useful framework for consensus clinical decision‐making [3]. MDT performance relies on the data included in the discussion. Among available diagnostic tools, endoscopic ultrasound (EUS) is an important adjunct to cross‐sectional imaging in PCL evaluation because it enables detailed assessment of cyst morphology as well as obtaining cyst fluid for cytologic, molecular, and biochemical analysis [4]. However, EUS is an invasive modality not routinely recommended for evaluation of all PCLs, and its incremental contribution to MDT discussions and clinic decision‐making regarding surgical candidacy has remained uncertain.

In this issue, Dbouk and colleagues address this question [5]. Using a multicenter, blinded, mock‐MDT crossover design, they evaluated 40 consecutive PCL cases, including 20 high‐risk lesions with advanced neoplasia and 20 low‐risk lesions defined by low‐grade dysplasia or stable long‐term surveillance. Four expert MDTs across two tertiary centers reviewed the same cases under three conditions: with cross‐sectional imaging alone, cross‐sectional imaging combined with upfront EUS data, or with EUS revealed after an initial decision had already been made without it. This elegant design allowed them not only to assess the added value of EUS, but also examine whether the timing of its introduction into the MDT discussion influenced interpretation.

The findings are noteworthy and key strengths include multi‐institutional design and structured evaluation of bias. When EUS was available upfront, MDTs more accurately identified neoplastic cysts, more reliably recognized PCLs harboring advanced neoplasia, and, most importantly, made more appropriate surgical referral decisions for PCLs with advanced neoplasia than when reviewing based on cross‐sectional imaging alone. The upfront incorporation of EUS data also reduced inappropriate referral of cysts without advanced neoplasia, although this difference wasn't statistically significant. In parallel analyses, MDT performance with upfront EUS also exceeded that of applying guideline‐based approaches in isolation.

One of the study's most interesting aspects is the crossover analysis. When MDTs first formed an impression without EUS and subsequently reviewed the EUS findings, performance declined compared to cases in which EUS was available from the outset. This was particularly evident for classification of cysts as neoplastic and for surgical decision‐making. The authors interpret this as evidence of cognitive bias, particularly anchoring or confirmation bias, whereby an early impression formed using cross sectional imaging in the same patient may shape the way subsequent data are incorporated. This observation is highly relevant, because it highlights that in complex diagnostic settings, the sequence in which information is presented may matter almost as much as the information itself.

However, as with any study, the findings should be interpreted in light of several important limitations. Because EUS is generally reserved for PCLs with worrisome or high‐risk features, diagnostic uncertainty, or other concerning clinical findings [6], the study cohort may represent a selected subgroup in whom the justification for advanced evaluation and potential need for resection was already evident. This distinction is clinically important, as nearly 80% of PCLs don't have worrisome or high‐risk features and hence don't require routine evaluation with EUS. Accordingly, these findings should not be interpreted as supporting upfront EUS for all PCLs. Rather, a more appropriate conclusion is that, when there is a clear clinical indication for EUS, performing it before MDT review may be advantageous by allowing the discussion to proceed with more complete diagnostic information. The results also underscore current approaches remain imperfect. Even in the best‐performing group, 15% of patients were incorrectly referred to surgery and the incorrect surgical intervention rate was not significantly different with or without EUS. This is not a trivial observation, particularly in a field where unnecessary pancreatic resection carries substantial morbidity and real‐world estimates of low‐grade PCL resections are higher than what was reported in this small selective cohort. Finally, it is important to recognize that this study was performed at two large referral centers where the expertise of the MDT may exceed what is available in more resource restricted healthcare settings.

Thus, while the authors suggest that MDT performance sets a high bar for future biomarkers, that bar should not discourage further innovation, especially development of biomarkers and imaging tools that can be more widely available, including settings with less robust clinical expertise and access to MDT. Future studies assessing MDT impact need to move beyond comparisons with guidelines or cyst fluid biomarkers alone, evaluate MDT performance in larger, diverse healthcare settings and assess whether next‐generation sequencing [7], emerging molecular cyst fluid or blood‐based biomarkers [8], contrast‐enhanced EUS, or AI‐based cyst imaging can further improve the precision of PCL management [9].

## Conflicts of Interest

Mayo Clinic and Exact Sciences have an intellectual property development agreement. S.M. is listed as inventor under this agreement and could share potential future royalties as an employee of Mayo Clinic.

## Data Availability

Data sharing not applicable to this article as no datasets were generated or analyzed during the current study.
